# Correlation of execution time of the walking test between force platform and BESTest in diabetic individuals

**DOI:** 10.1186/1758-5996-7-S1-A243

**Published:** 2015-11-11

**Authors:** Nathalia Cristina de Souza Borges, Ariane Hidalgo Mansano Pletsch, Fernanda Maria Ferreira da Cruz, Natalia Akemi Terada, Duany Maria Villar, Ana Laura Franzini Sutilo, Rinaldo Roberto de Jesus Guirro

**Affiliations:** 1 Faculdade De Medicina De Ribeirão Preto/Usp-Universidade De São Paulo, Ribeirão Preto, Brazil

## Background

Type-2 diabetes mellitus is considered a great problem of public health, resulting in complications such as deficits in functional performance of the lower limbs and falls consequently, which can interfere with the balance maintenance.

## Objective

To assess the gait of type-2 diabetic individuals by correlating the Balance Evaluation System Test (BESTest) for clinical balance evaluation on plane surface and the Walk Across test on Neurocom Balance Master Sistem® platform.

## Method

Forty-two type-2-diabetic male and female volunteers aged between 45 and 64 yrs. old were recruited for study, all with their glycaemic levels controlled and being physically active. Gait was assessed as follows: 1) gait test on plane surface for clinical balance evaluation (BESTest) and 2) Walk Across test on Neurocom Balance Master Sistem® platform at distances of 6 m and 1.5 m, respectively. Analyses: Data distribution was verified with the Shapiro-Wilk test and application of Pearson's correlation at significance level of 5%. Magnitude of the correlations was based on the Munro's classification (low: 0.26 to 0.49; moderate: 0.50 to 0.69; high: 0.70 to 0.89; very high: 090 to 1.00). For data processing, the SPSS software version 17.0 was used at significant level of 5%.

## Results

Forty-two diabetic male and female subjects with mean age of 55.2±6.4, mean height of 1.65±0.08m, and mean BMI of 80.64±13.9Kg were assessed. Significant positive and moderate correlations (r=0.574) were found between the times (in seconds) taken for the distances covered in the platform (2.43±0.44) and BESTest (6.32±1.33).

## Conclusion

Considering the correlation at execution time between BESTest and Balance Master Sistem® platform, we suggest the use of balance evaluation system test (BESTest) for clinical balance evaluation on plane surface instead of the Walk Across test, since the former is of low cost and ease to be applied, thus having broader range and more availability for gait assessment in the clinical practice. However, we emphasise that further studies on this theme should be carried out, since the correlation was found to be moderate.

